# Coincidence between Geographical Distribution of *Leptotrombidium scutellare* and Scrub Typhus Incidence in South Korea

**DOI:** 10.1371/journal.pone.0113193

**Published:** 2014-12-12

**Authors:** Jong Yul Roh, Bong Gu Song, Won Il Park, Eun Hee Shin, Chan Park, Mi-Yeoun Park, Kyu Sik Chang, Wook Gyo Lee, Hee Il Lee, E-Hyun Shin

**Affiliations:** 1 Division of Medical Entomology, Center for Immunology and Pathology, Korea National Institute of Health, Cheongju, Korea; 2 Division of Arboviruses, Center for Immunology and Pathology, Korea National Institute of Health, Cheongju, Korea; 3 Division of Biosafety Evaluation and Control, Korea National Institute of Health, Cheongju, Korea; University of Texas Medical Branch, United States of America

## Abstract

To clarify the geographical distribution of scrub typhus vectors in Korea, a survey of larval trombiculid mites was conducted from 2005 to 2007 by collecting wild small mammals twice a year (spring and autumn) at 24 sites nationwide. A total of 67,325 mites representing 4 genera and 14 species were collected from 783 trapped rodents, corresponding to a chigger index (number of chigger mites per rodent) of 86.0. The predominant mite species were *Leptotrombidium pallidum* (52.6%), *Leptotrombiduim scutellare* (27.1%), *Leptotrombidium palpale* (8.2%), *Leptotrombidium orientale* (5.6%), and *Neotrombicula tamiyai* (1.7%). However, the proportions of *L. scutellare* in southern areas, including endemic provinces such as Jeollabuk-Do (34.3%), Jeollanam-Do (49.0%), and Gyeongsangnam-Do (88%), were relatively higher than in central Korean regions where *L. pallidum* was predominant. In autumn, the ratio of *L. scutellare* increased to 42% while the ratio of *L. pallidum* decreased. The geographical distribution map of the *L. scutellare* chigger index was identical to the incidence pattern of scrub typhus, whereas those of overall mites and *L. pallidum* showed no relationship with case incidence patterns. Distribution mapping analysis shows an identical geographical distribution of *L. scutellare* and epidemic incidence of scrub typhus in South Korea. *L. pallidum* could be another vector at all other parts of the Korean peninsula, including the eastern and northern regions that have a low level of scrub typhus incidence.

## Introduction

Scrub typhus (tsutsugamushi disease) is caused by the rickettsial bacterium, *Orientia tsutsugamushi*, which are transmitted through bites from infected larval trombiculid mites [1]. *Orientia tsutsugamushi* is maintained in mite populations by transovarial transmission [2], [3]. 

In South Korea, scrub typhus is an acute febrile disease most common in the autumn months; the annual case number has fluctuated around 5,000 (approximately 10 per 100,000 persons) since 2004 [4]. As a reportable disease, confirmed cases of scrub typhus have been reported to Korea Centers for Disease Control and Prevention (KCDC) by the National Notifiable Disease Surveillance System since 1994. In 2013, 10,365 cases were reported nationwide; the highest number ever recorded, this total represents a 38.1-fold increase compared to 274 cases reported in 1995 [5]. Over 90% of cases were reported during the epidemic period, from October to November [4], [6].

Known vector species of chigger mites include *Leptotrombidium akamushi*, *L. pallidum*, *L. scutellare*, *L. deliense*, and *L. imphalum* in Japan, Taiwan, Thailand, and China, respectively [7], [8], [9], [10]. Ree et al. (1991) first reported *L. pallidum* as a vector in Korea, with an infection rate of 0.4% in 447 tested mites [11]. *L. scutellare* is another key vector, with an infection rate of 0.5% [12]. The *O. tsutsugamushi* bacterium has also been detected in *L. palpale*, *L. orientale*, *L. zetum*, *Neotrombicula japonica* and *Euschoengastia koreaensis* chigger mites [13], [14].

The distribution of chigger mites in Korea has been mainly surveyed in epidemic regions, such as Jeollanam-do, Gyeonggi-do, and Chungcheongnam-do [3], [15], [16]. A 2006 survey of 13 localities in Korea from October to November was the first nationwide survey [17].

In this study, we surveyed 24 localities nationwide in spring and autumn from 2005 to 2007 and analyzed the relationship between trombiculid mite geographical distribution and scrub typhus epidemic regions in Korea.

## Materials and Methods

### Surveillance localities and periods

**Figure 1:**
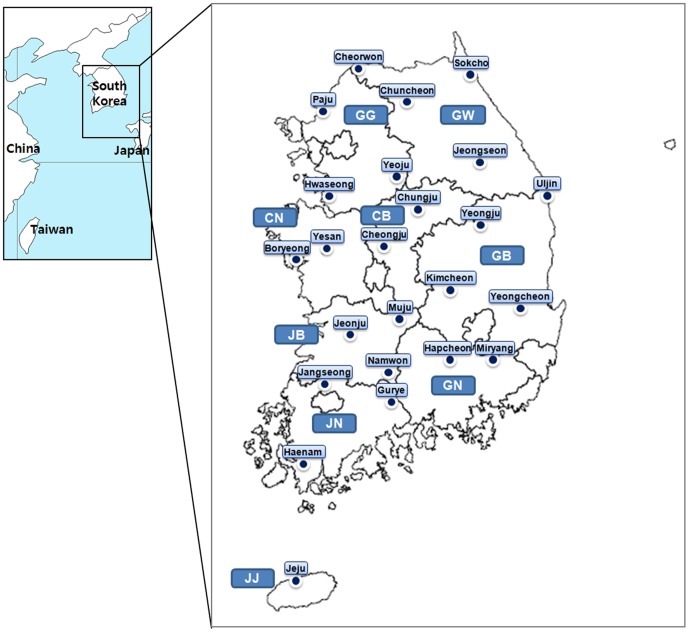
Scrub typhus surveillance in the Korean peninsula. Dots indicate nationwide surveillance locations. Each name indicates a city level locality (-Si, -Gun, and -Gu in the Korean administrative area system). Province abbreviations: GG, Gyeonggi-Do; GW, Gangwon-Do; CB, Chungcheongbuk-Do; CN, Chungcheongnam-Do; GB, Gyeongsangbuk-Do; GN, Gyeongsangnam-Do; JB, Jeollabuk-Do; JN, Jeollanam-Do; JJ, Jeju-Do.

Larval trombiculid mites were collected from wild rodents captured at 24 sites nationwide from 2005 to 2007 (Fig. 1 and S1 Table). Collections were performed at each location in spring (March–May) and autumn (October–November). Collection information, including locality, collection year and month, number of traps installed, and number of rodents captured, is summarized in S1 Table. Rodent trap install sites included rice fields, cropped fields, reservoirs, waterways, hillsides, grass fields and riversides.

### Collection of small mammals and chigger mites

The animal protocol used in this study was reviewed and approved based on ethical procedures and scientific care by the KCDC-Institutional Animal Care and Use Committee (KCDC-IACUC; KCDC-046-13-2A). There was no need for specific permission for each collecting site, because these sites were not located at national parks or protected areas. The selection of collecting sites was supported by each local Public Health Center. The collected rodents were not the endangered or protected species in Korea.

The collection of small mammals was performed at 24 collection sites nationwide. A total of 10 to 15 Sherman live folding traps (3×3×9 inches), baited with a peanut butter spread biscuit, were set up at five to seven points in the collection site at 3–5 m intervals and collected the next morning. Collected wild rodents were euthanized by compressed carbon dioxide (CO_2_). The wild rodent corpses were hung over glass bowls filled with tap water for 24 h to collect dropped chigger mites. Chigger mites were recovered from the water surface with a fine brush and stored at 4°C for further identification.

### Identification of chigger mites

Individual chigger mites were transferred to glass slides and mounted with PVA MTNG medium (Polyvinyl alcohol mounting medium, Bioquip). The specimens were identified to species level under stereo-microscopic examination using morphological keys [18].

### ArcGIS geographical analysis

**Figure 2:**
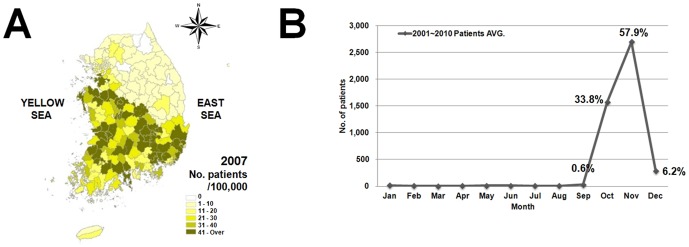
Scrub typhus incidence in 2007 (A), and average monthly number of patients with scrub typhus (B) from 2001 to 2010. The figure is generated using data from the National Notifiable Disease Web Statistics System (NNDWSS) of the KCDC. Percentage (%) indicates monthly incidence rate.

To compare the geographical distribution of vectors, distribution maps were drawn by interpolation using the IDW (Inverse Distance Weighted) technique among Spatial Analyst Tools in ArcGIS 9.0 (2004. Environmental Research Systems Institute, Redlands, CA, USA). Information about patients in South Korea diagnosed with scrub typhus in 2007 was obtained from the National Notifiable Disease Web Statistics System (NNDWSS) of the KCDC (Fig. 2A). Scrub typhus was diagnosed by indirect immune-fluorescent assay and nested polymerase chain reaction (PCR) by regional Institutes of Health and Environment and hospitals and reported to the NNDWSS of the KCDC.

## Results and Discussion

A total of 5,538 traps were installed and 783 wild rodents captured at 24 regions in 9 South Korean provinces (S1 Table). The overall trapping rate was 14.1; the highest trapping rate (30.5%) was recorded in riverside locations. Among trapped rodents, *Apodemus agrarius* was dominant in all regions, accounting for 87.4% of the collections (data not shown), followed by *Crocidura lasiura* and *Micromys minutus* at 8.0% and 4.2%, respectively. From those rodents, 67,325 mites representing 4 genera and 14 species were collected, with a chigger index (C.I., number of chigger mites per rodent) of 86.0 (Table 1). The number and C.I. (43,434 and 108.6) of chigger mites in autumn were 1.8-fold and 1.7-fold higher than in spring (23,891 and 62.4). This result suggests that the high density of chigger mites may affect the high incidence rate (92.3%) of scrub typhus in autumn. In South Korea, the incidence of scrub typhus is generally highest during autumn (over 90%), from September to November, while the incidence in other seasons (spring, summer and winter) ranged from 0.1 to 0.3% (Fig. 2B). However, the difference in chigger indices between spring and autumn does not proportionally explain the difference in scrub typhus incidence in the same seasons.

**Table 1:** Species of chigger mites from small mammals collected through the nationwide survey in South Korea.

Locality	S/A^a^	No. of captured rodent	No. of chigger mites	Chigger Index (C.I.)	*C. ikaoensis*	*E. korea-ensis*	*L. gemiti-culum*	*L. hiranu-mai*	*L. orientale*		*L. pallidum*	*L. palpale*	*L. scutellare*	*L. subinter-medium*	*L. zetum*	*N. gardellai*	*N. japonica*	*N. kwangn-engensis*	*N. tamiyai*
**GG**		**78**	**7,110**	**91.2**	**10**	**21**	**-**	**-**	**427**		**5,237**	**839**	**131**	**-**	**232**	**2**	**11**	**-**	**200**
Hwaseong	S	8	404	50.5	-	-	-	-	113		166	68	-	-	57	-	-	-	-
	A	17	1,410	82.9	-	9	-	-	47		741	427	131	-	41	-	11	-	2
Yeoju	S	17	2,951	173.6	10	-	-	-	166		2,640	67	-	-	38	-	-	-	31
	A	13	102	7.8	-	-	-	-	26		70	4	-	-	-	2	-	-	-
Paju	S	21	2,243	106.8	-	12	-	-	75		1,620	273	-	-	96	-	-	-	167
	A	2	-	0.0	-	-	-	-	-		-	-	-	-	-	-	-	-	-
**GW**		**111**	**9,424**	**84.9**	**4**	**399**	**382**	**22**	**765**		**5,855**	**671**	**14**	**-**	**26**	**2**	**616**	**476**	**193**
Chuncheon	S	13	798	61.4	-	-	-	-	144		548	84	-	-	2	-	-	-	21
	A	15	80	5.3	-	-	-	-	4		69	2	-	-	-	-	-	4	-
Sokcho	S	11	271	24.6	-	-	-	-	123		106	42	-	-	-	-	-	-	-
	A	31	2,517	81.2	-	-	-	-	16		1,349	443	14	-	-	-	248	421	26
Jeongseon	S	18	242	13.4	-	-	-	-	2		185	28	-	-	-	-	-	-	28
	A	3	84	28.0	-	59	-	-	10		-	10	-	-	-	-	-	-	5
Cheorwon	S	11	2,691	244.6	-	-	-	22	373		2,141	36	-	-	20	-	-	2	97
	A	9	2,741	304.6	4	340	382	-	93		1,457	26	-	-	4	2	368	49	16
**CB**		**68**	**6,893**	**101.4**	**10**	**232**	**44**	**-**	**102**		**5,146**	**284**	**980**	**-**	**20**	**37**	**12**	**20**	**6**
Cheongju	S	18	688	38.2	-	4	-	-	6		531	146	-	-	-	-	-	-	2
	A	26	3,056	117.5	10	163	-	-	14		1,712	89	980	-	18	37	8	20	4
Chungju	S	18	2,906	161.4	-	-	-	-	-		2,859	47	-	-	-	-	-	-	-
	A	6	243	40.5	-	66	44	-	82		44	2	-	-	2	-	4	-	-
**CN**		**50**	**4,288**	**85.8**	**-**	**37**	**-**	**-**	**638**		**2,500**	**284**	**792**	**-**	**34**	**-**	**2**	**-**	**-**
Boryeong	S	17	781	45.9	-	-	-	-	184		539	46	-	-	12	-	-	-	-
	A	16	806	50.4	-	-	-	-	5		504	188	109	-	-	-	-	-	-
Yesan	S	7	867	123.9	-	6	-	-	310		521	30	-	-	-	-	-	-	-
	A	10	1,834	183.4	-	31	-	-	139		936	20	683	-	22	-	2	-	-
**JB**		**104**	**8,700**	**83.7**	**7**	**67**	**-**	**-**		**263**	**4,283**	**770**	**2,986**	**2**	**17**	**2**	**19**	**5**	**279**
Jeonju	S	13	270	20.8	-	-	-	-		54	214	2	-	-	-	-	-	-	-
	A	6	1,207	201.2	-	49	-	-		79	382	20	675	-	-	2	-	-	-
Namwon	S	23	1,588	69.0	2	-	-	-		51	1,106	167	-	-	-	-	-	-	262
	A	17	2,196	129.2	3	10	-	-		22	580	66	1,495	-	3	-	-	5	12
Muju	S	18	325	18.1	-	-	-	-		2	286	32	-	2	-	-	-	-	3
	A	27	3,114	115.3	2	8	-	-		55	1,715	483	816	-	14	-	19	-	2
**JN**		**126**	**17,251**	**136.9**	**10**	**23**	**-**	**-**		**977**	**5,741**	**1,415**	**8,449**	**6**	**90**	**2**	**30**	**13**	**497**
Gurye	S	18	1,689	93.8	-	-	-	-		198	1,470	12	1	2	6	-	-	-	-
	A	9	1,374	152.7	-	9	-	-		70	392	44	837	4	4	-	-	13	2
Haenam	S	9	144	16.0	-	-	-	-		120	-	14	3	-	8	-	-	-	-
	A	32	6,370	199.1	10	12	-	-		404	12	372	5,519	-	10	2	30	-	-
Jangseong	S	28	1,288	46.0	-	2	-	-		95	943	59	-	-	54	-	-	-	135
	A	30	6,386	212.9	-	-	-	-		90	2,924	915	2,090	-	8	-	-	-	359
**GB**		**167**	**8,112**	**48.6**	**20**	**74**	**-**	**-**		**318**	**6,628**	**970**	**28**	**-**	**43**	**6**	**13**	**11**	**2**
Kimcheon	S	23	2,410	104.8	-	-	-	-		-	2,364	46	-	-	-	-	-	-	-
	A	33	2,968	89.9	-	-	-	-		-	2,188	739	25	-	14	-	2	-	-
Yeongju	S	25	1,014	40.6	2	2	-	-		270	721	16	-	-	2	-	-	-	2
	A	5	355	71.0	-	-	-	-		15	325	2	-	-	-	-	2	11	-
Yeongcheon	S	28	28	1.0	2	1	-	-		-	-	20	-	-	4	-	-	-	-
	A	17	242	14.2	14	64	-	-		6	15	108	3	-	23	-	9	-	-
Uljin	S	4	140	35.0	-	-	-	-		21	119	-	-	-	-	-	-	-	-
	A	32	955	29.8	2	7	-	-		6	896	39	-	-	-	6	-	-	-
**GN**		**51**	**4,753**	**93.2**	**-**	**2**	**-**	**-**		**288**	**27**	**251**	**4,183**	**-**	**1**	**-**	**-**	**-**	**1**
Miryang	S	11	25	2.3	-	-	-	-		9	11	3	2	-	-	-	-	-	-
	A	16	4,151	259.4	-	-	-	-		167	16	84	3,884	-	-	-	-	-	-
Hapcheon	S	11	80	7.3	-	-	-	-		75	-	4	-	-	1	-	-	-	-
	A	13	497	38.2	-	2	-	-		37	-	160	297	-	-	-	-	-	1
**JJ**		**28**	**794**	**28.4**	**-**	**24**	**-**	**-**		**13**	**-**	**13**	**667**	**-**	**78**	**-**	**-**	**-**	**-**
Jeju	S	13	48	3.7	-	2	-	-		2	-	10	1	-	34	-	-	-	-
	A	15	746	49.7	-	22	-	-		11	-	3	666	-	44	-	-	-	-
**Total**	S	383	23,891	62.4	16	29	-	22		2,393	19,090	1,252	7	4	334	-	-	2	748
	A	400	43,434	108.6	45	851	426	-		1,398	16,327	4,246	18,224	4	207	51	703	523	429
		**783**	**67,325**	**86.0**	**61**	**880**	**426**	**22**		**3,791**	**35,417**	**5,498**	**18,231**	**8**	**541**	**51**	**703**	**525**	**1,177**

a S or A indicates spring season or autumn season.

**Figure 3:**
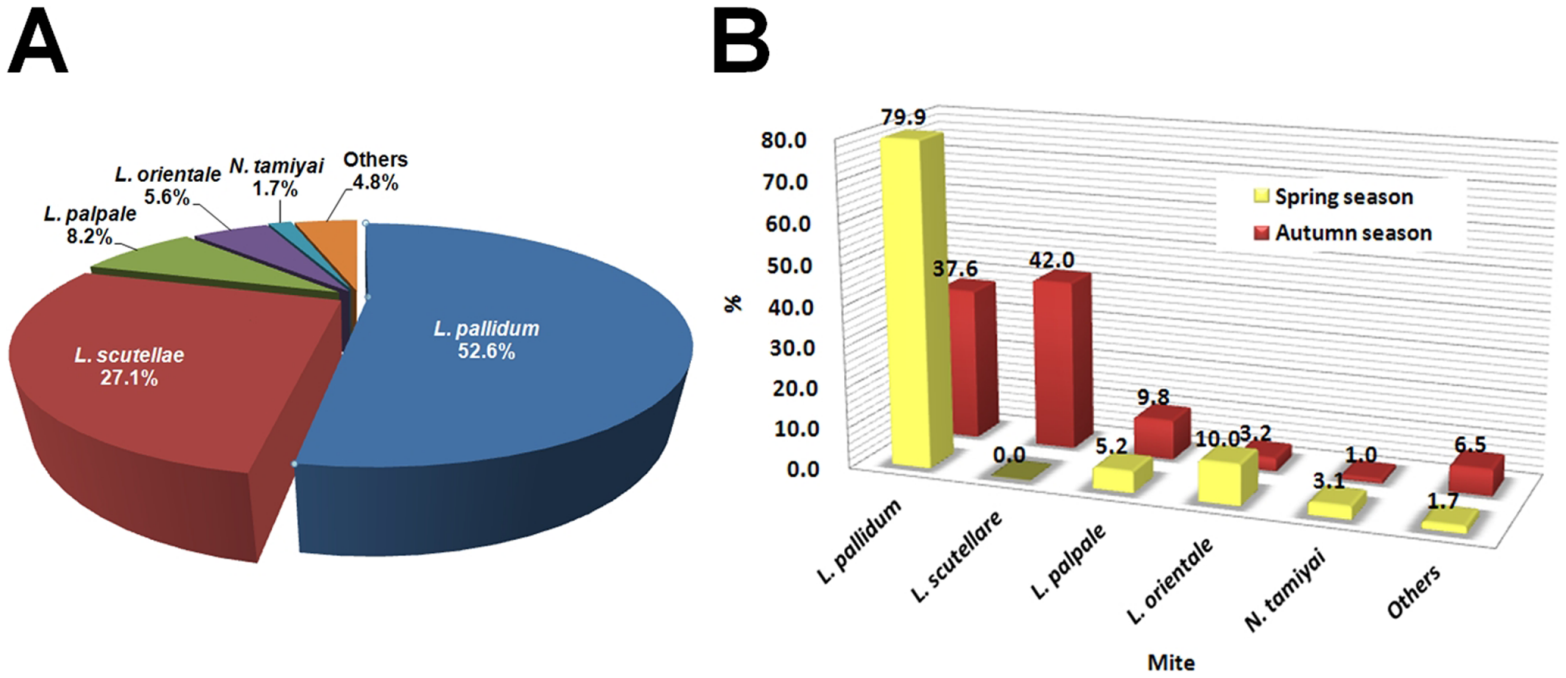
Total percentages (A) and seasonal prevalence (B) of chigger mites collected through Korean national surveys between 2005 and 2007. “Others” indicates 9 minor species including *E. koreaensis*.

The predominant species identified in this study were *Leptotrombidium pallidum* (52.6%), *L. scutellare* (27.1%), *L. palpale* (8.2%), *L. orientale* (5.6%), and *Neotrombicula tamiyai* (1.7%) (Fig. 3A). This finding is similar to a previous report [17]: Lee *et al.* (2009) found *L. pallidum* to be the dominant species (74.9%), followed by *L. scutellare* (18.9%) and *L. palpale* (2.7%). Lee et al. further showed that *L. pallidum* and *L. scutellare* are the predominant scrub typhus vectors, accounting for approximately 90% of the chigger mite population in Korea. In autumn, the ratio of *L. scutellare* increased to 42.0% while *L. pallidum* decreased to 37.6% (Fig. 3B). *L. palpale* density also increased 5.2% to 9.8% in autumn. The increased density of *L. scutellare* and *L. palpale* may affect the high autumn incidence of scrub typhus (Fig. 3B). In Korea, spring is the only seeding season, while autumn is the main harvest season. Therefore, the high *L. pallidum* population without corresponding scrub typhus case increases in spring may be explained by seasonal differences in human behavior patterns that limit human exposure to *L. pallidum* in spring.

The highest C.I. was recorded in Jeollanam-Do (JN, 136.9) among 9 provinces, followed by Chungcheongbuk-Do (CB, 101.4), and Gyeongsangnam-Do (GN, 93.2) (Table 1). However, the regions with the highest average incidence over three years (2005 to 2007) were Jeollabuk-Do (JB), Chungcheongnam-Do (CN) and Jeollanam-Do (JN). The prevalence of *L. scutellare* in southern areas, including endemic provinces such as Jeollabuk-Do (2,986, 34.3%), Jeollanam-Do (8,449, 49.0%), and Gyeongsangnam-Do (4,183, 88%) was relatively higher than in the central areas where *L. pallidum* was predominant.

**Figure 4:**
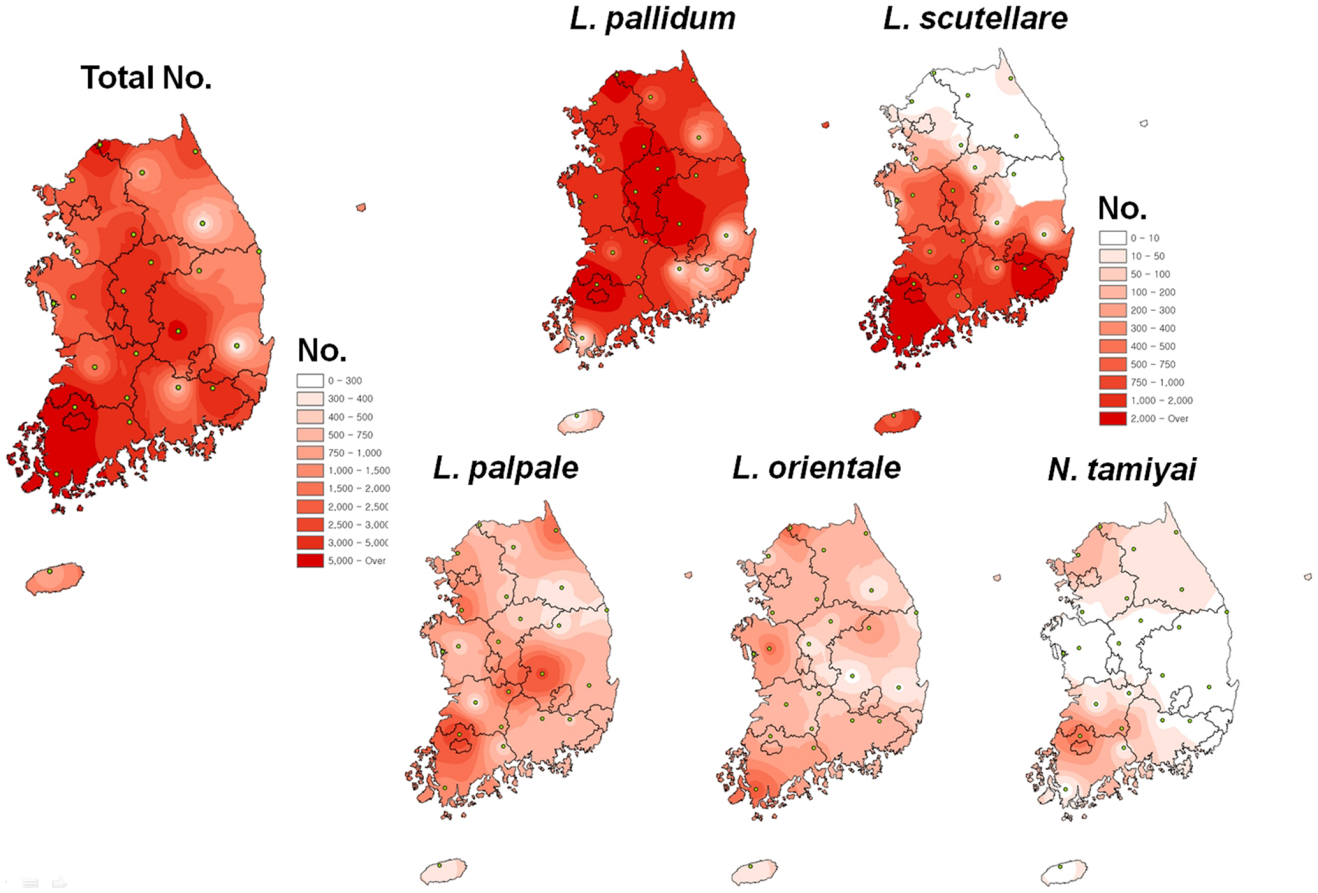
Geographical distribution of chigger mites. The map color indices differ between the total number (0– over 5,000) and species maps (0– over 2,000).

To visualize the geographical distribution of chigger mites in South Korea, we analyzed collection data using the interpolation method of the IDW tool in the ArcGIS program. We first drew distribution maps based on the number of chigger mites for the five predominant species (Fig. 4). *L. pallidum*, *L. palpale* and *L. orientale* were evenly distributed nationwide. However, *L. scutellare* was found in the western and southern parts of nation and *N. tamiyai* was not observed in central Korea. Interestingly, of the five species, the distribution pattern of *L. scutellare* was very similar to regions prevalent for scrub typhus (Fig. 2A and Fig. 4).

**Figure 5:**
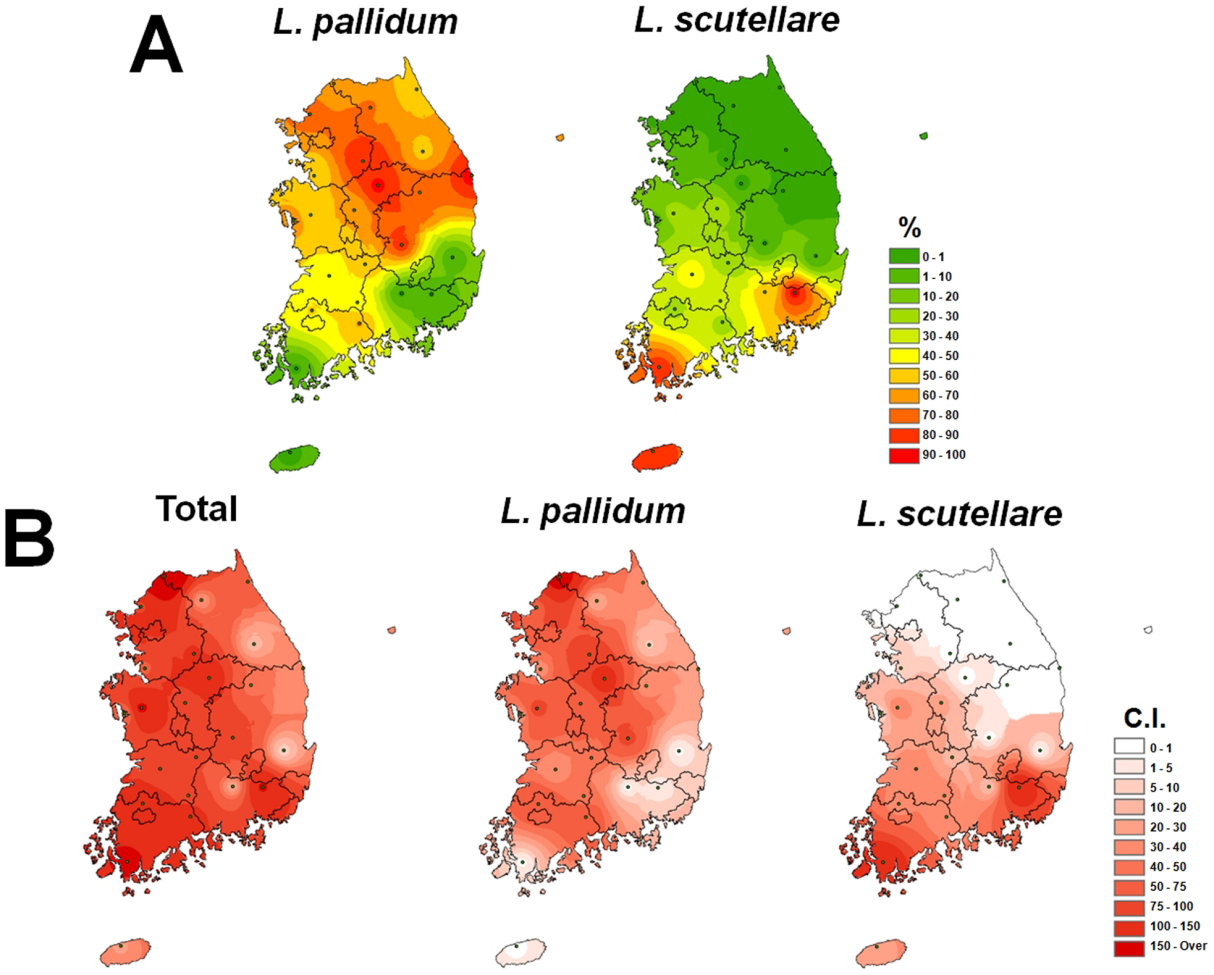
Geographical distribution based on percentages (A) and C.I. (B) of *L. pallidum* and *L. scutellare*. Chigger index (C.I.) indicates the number of chigger mites on one small mammal.

For regional analysis of predominant species, the percentages of *L. pallidum* and *L. scutellare* were also mapped (Fig. 5A). *L. pallidum* accounted for over 50% of mites collected in northern and central regions of Korea, while *L. scutellare* represented 30% of mites in the western and southern parts (Fig. 5A). In addition, the map of *L. scutellare* C.I. showed a similar pattern to the prevalence of scrub typhus in South Korea (Fig. 2A and Fig. 5B). Ree et al. (1991) first reported *L. pallidum* as a vector in Korea, with an infection rate of 0.4% in 447 tested mites [11]. *L. scutellare* is also known to be another key vector, with an infection rate of 0.5% [12]. *O. tsutsugamushi* bacteria have also been detected in *L. palpale*, *L. orientale*, *L. zetum*, *Neotrombicula japonica* and *Euschoengastia koreaensis* mites [13], [14]. According to Lee et al. (2011), the infection rates of *O. tsutsugamushi* did not differ significantly among vector mite species (range: 1.5–5.3%); infections rates in *L. pallidum* and *L. scutellare* were 1.5% and 3.7%, respectively, and the highest rate (5.3%) was in *L. palpale* [14]. *L. pallidum* and *L. scutellare* are considered major scrub typhus vectors in Korea. Scrub typhus is most prevalent in the western and southern regions of the Korean peninsula (Chungcheongnam-Do (CN), Jeollabuk-Do (JB), Jeollanam-Do (JN), and Gyeongsangnam-Do (GN)). The distribution and C.I. map of *L. scutellare* was identical to the incidence pattern of scrub typhus, whereas the C.I. maps *L. pallidum* and chigger mites overall show no relationship with the incidence pattern (Fig.4 and Fig. 5B). However, the *L. scutellare* C.I. in the Haenam region did not clearly match the scrub typhus incidence pattern; further study of this region is necessary. In conclusion, our distribution mapping analysis suggests that the geographical distribution of *L. scutellare* is identical to the epidemic incidence of scrub typhus in South Korea. Additionally, *L. pallidum* could be another vector in the Korean peninsula, including eastern and northern regions.

## Supporting Information

S1 Table **Collection sites of small mammals in Korean peninsula.**
(DOC)
