# Supplementary material for: Coincidence between Geographical Distribution of Leptotrombidium scutellare and Scrub Typhus Incidence in South Korea
Source: PLoS One. 2014 Dec 12;9(12):e113193. doi: 10.1371/journal.pone.0113193 (PMC4264734; doi:10.1371/journal.pone.0113193)
Supplement: S1 Table — Collection sites of small mammals in Korean peninsula. (DOC) [file pone.0113193.s001.doc]

Table S1. Collection sites of small mammals in Korean peninsula

| Locality | | | Season(S/A^a^) | Collection date | | | | No. of installed trap | No. of captured rodent | Site abbreviation |
| --- | --- | --- | --- | --- | --- | --- | --- | --- | --- | --- |
|  |  |  |  | Year | | | Month |  |  |  |
| **Gyeonggi-Do** | | |  |  | | |  | **579** | **78** | **GG** |
|  | Hwaseong | | S | 2006 | | | Mar | 122 | 8 | GG-HS |
|  |  | | A | 2005 | | | Nov | 117 | 17 |  |
|  | Yeoju | | S | 2006 | | | Apr | 110 | 17 | GG-YJ |
|  |  | | A | 2005 | | | Oct | 60 | 13 |  |
|  | Paju | | S | 2006 | | | Apr | 110 | 21 | GG-PJ |
|  |  | | A | 2005 | | | Oct | 60 | 2 |  |
| **Gangwon-Do** | | |  |  | | |  | **715** | **111** | **GW** |
|  | Chuncheon | | S | 2006 | | | Apr | 80 | 13 | GW-CC |
|  |  | | A | 2005 | | | Oct | 75 | 15 |  |
|  | Sokcho | | S | 2006 | | | Apr | 109 | 11 | GW-SC |
|  |  | | A | 2005 | | | Nov | 95 | 31 |  |
|  | Jeongseon | | S | 2006 | | | Apr | 110 | 18 | GW-JS |
|  |  | | A | 2005 | | | Nov | 75 | 3 |  |
|  | Cheorwon | | S | 2006 | | | Apr | 105 | 11 | GW-CW |
|  |  | | A | 2005 | | | Oct | 76 | 9 |  |
| **Chungcheongbuk-Do** | |  | | |  | | | **410** | **68** | **CB** |
|  | Cheongju | | S | 2007 | | | May | 115 | 18 | CB-CeJ |
|  |  | | A | 2006 | | | Oct | 110 | 26 |  |
|  | Chungju | | S | 2006 | | | Apr | 110 | 18 | CB-CuJ |
|  |  | | A | 2005 | | | Oct | 75 | 6 |  |
| **Chungcheongnam-Do** | |  | | |  | | | **661** | **50** | **CN** |
|  | Boryeong | | S | 2006 | | | Apr | 110 | 17 | CN-BR |
|  |  | | A | 2005 | | | Nov | 71 | 16 |  |
|  | Yesan | | S | 2007 | | | Apr | 240 | 7 | CN-YS |
|  |  | | A | 2007 | | | Oct | 240 | 10 |  |
| **Jeollabuk-Do** | | |  |  | | |  | **678** | **104** | **JB** |
|  | Jeonju | | S | 2007 | | | Apr | 135 | 13 | JB-JJ |
|  |  | | A | 2007 | | | Oct | 135 | 6 |  |
|  | Namwon | | S | 2007 | | | Mar | 105 | 23 | JB-NW |
|  |  | | A | 2007 | | | Oct | 108 | 17 |  |
|  | Muju | | S | 2007 | | | Apr | 95 | 18 | JB-MJ |
|  |  | | A | 2007 | | | Oct | 100 | 27 |  |
| **Jeollanam-Do** | | |  |  | | |  | **715** | **126** | **JN** |
|  | Gurye | | S | 2006 | | | Apr | 110 | 18 | JN-GR |
|  |  | | A | 2005 | | | Nov | 110 | 9 |  |
|  | Haenam | | S | 2007 | | | Mar | 130 | 9 | JN-HN |
|  |  | | A | 2007 | | | Nov | 120 | 32 |  |
|  | Jangseong | | S | 2007 | | | Mar | 135 | 28 | JN-JS |
|  |  | | A | 2007 | | | Nov | 110 | 30 |  |
| **Gyeongsangbuk-Do** | | |  |  | | |  | **1026** | **167** | **GB** |
|  | Kimcheon | | S | 2007 | | | Apr | 110 | 23 | GB-KC |
|  |  | | A | 2007 | | | Oct | 105 | 33 |  |
|  | Yeongju | | S | 2007 | | | Apr | 130 | 25 | GB-YJ |
|  |  | | A | 2006 | | | Oct | 221 | 5 |  |
|  | Yeongcheon | | S | 2007 | | | Apr | 115 | 28 | GB-YC |
|  |  | | A | 2007 | | | Oct | 110 | 17 |  |
|  | Uljin | | S | 2007 | | | Apr | 115 | 4 | GB-UJ |
|  |  | | A | 2007 | | | Oct | 120 | 32 |  |
| **Gyeongsangnam-Do** | | |  |  | | |  | **504** | **51** | **GN** |
|  | Miryang | | S | 2007 | | | Apr | 120 | 11 | GN-MR |
|  |  | | A | 2007 | | | Nov | 115 | 16 |  |
|  | Hapcheon | | S | 2007 | | | Mar | 134 | 11 | GN-HC |
|  |  | | A | 2006 | | | Nov | 135 | 13 |  |
| **Jeju-Do** | | |  |  | | |  | **250** | **28** | **JJ** |
|  | Jeju | | S | 2007 | | | Mar | 125 | 13 | JJ-JJ |
|  |  | | A | 2007 | | | Nov | 125 | 15 |  |
| Total | 24 sites | |  |  | |  | | 5,538 | 783 |  |

^a^ S or A indicates spring season or autumn season.
